# Effect of *Poria cocos* on Puromycin Aminonucleoside-Induced Nephrotic Syndrome in Rats

**DOI:** 10.1155/2014/570420

**Published:** 2014-08-07

**Authors:** So Min Lee, Yun Jung Lee, Jung Joo Yoon, Dae Gill Kang, Ho Sub Lee

**Affiliations:** ^1^College of Oriental Medicine and Professional Graduate School of Oriental Medicine, Wonkwang University, Shinyong-dong, Iksan, Jeonbuk 570-749, Republic of Korea; ^2^Hanbang Body-Fluid Research Center, Wonkwang University, Shinyong-dong, Iksan, Jeonbuk 570-749, Republic of Korea; ^3^Brain Korea (BK) 21 plus team, Professional Graduate School of Oriental Medicine, Wonkwang University, Shinyong-dong, Iksan, Jeonbuk 570-749, Republic of Korea

## Abstract

Nephrotic syndrome is associated with altered renal handling of water and sodium and changes in the levels of aquaporins (AQPs) and epithelial Na channels (ENaCs). The dried sclerotia of *Poria cocos* Wolf (WPC) have been used for treating chronic edema and nephrosis. We evaluated the effects of WPC on puromycin aminonucleoside- (PAN-) induced renal functional derangement and altered renal AQP2 and ENaC expression. In the nephrotic syndrome rat model, animals were injected with 75 mg/kg PAN and then treated with Losartan (30 mg*·*kg^−1^ *·*day^−1^) or WPC (200 mg*·*kg^−1^ *·*day^−1^) for 7 days. In the WPC group, proteinuria and ascites improved significantly. Plasma levels of triglyceride, total cholesterol, and low-density lipoprotein- (LDL-) cholesterol reduced significantly in the WPC group. In addition, the WPC group exhibited attenuation of the PAN-induced increase in AQP2 and ENaC *α*/*β* subunit protein and mRNA levels. WPC suppressed significantly PAN-induced organic osmolyte regulators, reducing serum- and glucocorticoid-inducible protein kinase (Sgk1) and sodium-myo-inositol cotransporter (SMIT) mRNA expression. Our results show that WPC improves nephrotic syndrome, including proteinuria and ascites, through inhibition of AQP2 and ENaC expression. Therefore, WPC influences body-fluid regulation via inhibition of water and sodium channels, thereby, improving renal disorders such as edema or nephrosis.

## 1. Introduction

Treatment with puromycin aminonucleoside (PAN), generally used as a model for nephrotic syndrome, is characterized by an increase of mesangial matrix in the glomeruli leading to massive proteinuria, generalized edema, and hyperlipidemia [1–3]. This experimental model mimics minimal change glomerulopathy in human pathology. Nephrotic syndrome is associated with deranged renal water and sodium handling [4], such that the renal diluting function is decreased. However, the mechanism of PAN has not been yet elucidated.

The aquaporins (AQPs) are a family of hydrophobic integral transmembrane channel proteins that play a central role in renal epithelial water transport [5]. AQP2 is involved in chronic/adaptational control of body water balance in water channels exclusively expressed in the principal cells of the connecting tubule and collecting duct. According to a recent study, acute regulation involves vasopressin-induced trafficking of AQP2. AQP3 and AQP4 are present in the basolateral plasma membrane of collecting duct principal cells and represent exit pathways for water reabsorbed apically via AQP2 [6]. Furthermore, recent studies have shown that PAN-induced nephrotic syndrome involves apical targeting of renal epithelial Na channel (ENaC) subunit expression. It has been shown that in PAN-induced nephrotic syndrome sodium reabsorption is specifically increased in the collecting duct. This study explores the role of increased sodium reabsorption caused by dysregulation of the key sodium channels and transporters in the collecting duct.

The activity of the Na^+^/K^+^ pump in the cortical collecting duct (CCD) increases in experimental models of nephrotic syndrome [7, 8]. Thus, at the molecular level, sodium retention in the PAN nephrotic syndrome model is associated with Na^+^/K^+^-ATPase upregulation and increased targeting of ENaCs in the collecting duct. Regulators of ENaC abundance include aldosterone [9], vasopressin [10], and angiotensin II [11]. This channel is primarily expressed in the apical membranes of cells where sodium channels are involved in the maintenance of extracellular fluid volume and blood pressure, such as in the distal nephron [8, 12, 13]. The channel is composed of three homologous subunits, namely, *α*, *β*, and *γ* ENaC [14, 15], and the functional ENaC proteins are detected in the late distal convoluted tubule, connecting tubule (CNT), CCD, outer medullary collecting duct (OMCD), and, to a lesser extent, the inner medullary collecting duct (IMCD) [16]. The *α*-ENaC subunit is localized mainly in a zone in the apical domains, whereas the *β*- and *γ*-ENaC subunits are found throughout the cytoplasm [17].

In the previous study,* Poria cocos* Wolf (Chinese Pinyin: Fu Ling, WPC) markedly inhibited AQP2 expression in response to hypertonic stress* in vitro* [18]; however, the possible benefits and mechanism of this have not been fully elucidated in nephrotic syndrome animal models. In this study, we used the same WPC that was used in the previous study. The dried sclerotia of WPC are well-known oriental medicinal fungi that grow around the roots of pine trees in China, Japan, Korea, and North America. They exhibit some pharmacological characteristics and are widely used in traditional medicines. WPC, alone or in combination with other herbs, is often used to treat diabetes as well as other disorders [19, 20]. Specially, WPC has been known to have a diuretic effect and is used for the treatment of chronic edema and nephrosis [21, 22]. Several triterpenes, pachyman, and pachymaran have been identified from WPC [20, 23].

The aim of the present study was to determine the effects of WPC* in vivo* and to research its effects on ascites, proteinuria, plasma lipids, and change in water or epithelial sodium channels in a PAN-induced nephrotic syndrome model in rats.

## 2. Materials and Methods

### 2.1. Experimental Animals

The animal procedures were approved by the Institutional Animal Care and Utilization Committee for Medical Science of Wonkwang University. Male Sprague-Dawley rats (160 g) were purchased from Samtako, Inc. (Osan, Republic of Korea). All rats were housed individually in metabolic cages to allow urine collection for monitoring protein excretion. They were given free access to food and water. Food and water consumption was measured daily. In addition, the rats were weighed daily.

Rats received 75 mg/kg PAN (Santa Cruz Biotechnology, Inc., CA) or saline intraperitoneally and were divided into five groups: Sham (saline, *n* = 5), PAN treatment (PAN, *n* = 5), Los (PAN, Losartan 30 mg*·*kg^−1^ *·*day^−1^, *n* = 5), and WPC (PAN, WPC 200 mg*·*kg^−1^ *·*day^−1^, *n* = 5) for 7 days. At the end of the experiments, all rats were killed using a guillotine, and the blood and ascites were collected. The blood samples were centrifuged at 600 ×g for 15 min at 4°C, and plasma samples were separated. The weight of the ascites was measured by inserting a weighed tissue paper into the abdomen to absorb the ascites and then reweighing it.

### 2.2. Monitoring of Renal Function

Rats from each group were maintained in separate metabolic cages during all experiments, allowing quantitative urine collection and measurement of water and food intake. Urine samples were collected for the determination of creatinine level, osmolality, proteinuria, and other parameters of renal function. Furthermore, the creatinine levels in the plasma were measured using a colorimetric method with a spectrophotometer (Milton Roy, Rochester, NY). Ion concentrations were measured using an electrolyte analyzer (NOVA 5^+^, Biochemical, Waltham, MA) and osmolality was measured using an Advanced Cryomatic Osmometer (Advanced Instruments, Norwood, MS).

### 2.3. Biochemical Analysis of Plasma

On the day of sacrifice, trunk blood was collected in prechilled tubes containing 1 mg/mL ethylenediaminetetraacetic acid (EDTA) to measure plasma chemicals. The total cholesterol (T-Cho), high-density lipoprotein- (HDL-) cholesterol, low-density lipoprotein- (LDL-) cholesterol, triglyceride (TG), blood urea nitrogen (BUN), total bilirubin (T-Bil), total protein (T-Pro), and glucose levels in plasma were enzymatically measured using commercially available kits (ARKRAY, Inc., Minami-ku, Kyoto, Japan).

### 2.4. Protein Preparation and Western Blot Analysis

The renal medulla was homogenized with a buffer containing 250 mM sucrose, 10 mM triethanolamine, 10 mM acetic acid, 1 mM EDTA, 1 mM phenylmethylsulfonyl fluoride (PMSF), 1 mM benzamidine hydrochloride hydrate, and 1 mM dithiothreitol, at pH 7.4. The homogenate was then centrifuged at 600 ×g for 10 min at 4°C, and the supernatant was centrifuged at 1000 ×g for 5 min at 4°C. The proteins were separated using 10% SDS-polyacrylamide gel electrophoresis and transferred to a nitrocellulose membrane. Blots were then washed with TBST [10 mM Tris-HCl (pH 7.6), 150 mM NaCl, and 0.05% Tween-20], blocked with 5% bovine serum albumin (BSA) in TBST for 1 h, and incubated with the appropriate primary antibody at dilutions recommended by the supplier. The membrane was then washed and the primary antibodies were detected using goat anti-rabbit-IgG or anti-mouse-IgG conjugated to horseradish peroxidase, before the bands were visualized using enhanced chemiluminescence (Amersham, Buckinghamshire, UK). Protein expression levels were determined by analyzing the signals captured on the nitrocellulose membranes using the ChemiDoc image analyzer (Bio-Rad, Hercules, CA).

### 2.5. Membrane Protein Extraction

A tissue membrane fraction was prepared using Mem-PER (PIERCE, Rockford, IL), according to the manufacturer's instructions as described by Fukuchi et al. [24]. Briefly, the renal medulla was homogenized with reagent A on ice. The tube was mixed thoroughly using a vortex mixer for 10 s and then incubated for 10 min on ice. Diluted reagent B and reagent C were added to the lysed tissue. Samples were incubated at 4°C for 30 min and centrifuged at 10,000 ×g for 3 min. The supernatant was incubated for 10 min at 37°C (to separate the membrane protein fraction) and centrifuged at 10,000 ×g for 2 min (to isolate the hydrophobic fraction from the hydrophilic fraction). The bottom layer was used as the membrane extract.

### 2.6. Ribonucleic Acid (RNA) Isolation and Reverse Transcription Polymerase Chain Reaction (RT-PCR) Assay

Total RNA was isolated from cultured mIMCD-3 using a commercially available kit. The yield and purity of the RNA were confirmed by measuring the ratio of the absorbance at 260 and 280 nm. In the first step, cDNA was prepared from 500 ng RNA by reverse transcription in a final volume of 20 mL in an Opticon MJ Research instrument. The samples were incubated at 37°C for 60 min and 94°C for 5 min. The following sets of primers were used in the PCR amplification: AQP1, sense: 5′-GTC CCA CAT GGT CTA GCC TTG TCT G-3′, anti-sense: 5′-GGG AAG GGT CCT GGA GGT AAG TCA-3′; AQP2, sense: 5′-GCC CCT TGC AGG AAC CAG ACA-3′, anti-sense: 5′-GCC AAA GCG GGA ATG ACA GTC-3′; AQP3, sense: 5′-GGC TAA AAA CGC TCC CTG TAT CCA-3′, anti-sense: 5′-GGA GTT TCC CAC CCC TAT TCC TAA A-3′; SMIT, sense: 5′-CAC TGT GAG TGG ATA CTT CC-3′, anti-sense: 5′-TCT CTT AAC TTC CTC AAA CC-3′; Sgk1, sense: 5′-GGA ACA CAG CCG AGA TGT ATG ACA A-3′, anti-sense: 5′-AAC TGC TTC CGC GGC TTC TTT CAC AC-3′; MR, sense: 5′-ACA GCT CAC CCC TAC CT TGG T-3′, anti-sense: 5′-CTT GAC GCC CAC CTA ACA TGT-3′; *α*-ENaC, sense: 5′-ACT GAA CTG TGC TCA GGG ATG A-3′, anti-sense: 5′-ATC TGC CTA CCT GGT CCA AGT G-3′; *β*-ENaC, sense: 5′-GGC TAG AGT CCT CTC CTG GTT-3′, anti-sense: 5′-GCC AAC TAG GGC AAG GAT TCT T-3′; *γ*-ENaC, sense: 5′-CTC ATT GTG CCA TGT GCC TCT A-3′, anti-sense: 5′-TAA TTT CTT CTG TCA CCC CAT CAG-3′; GAPDH, sense: 5′-TCA CCA TCT TCC AGG AGC GAG-3′, anti-sense: 5′-AAG GTG CAG AGA TGA TGA CCC TC-3′.

### 2.7. Quantitative Real-Time PCR

A total RNA reverse transcriptase reaction (Superscript III, Invitrogen, Carlsbad, CA) was performed. Total RNA amplification was performed using a two-step procedure as described in the SYBR RT-PCR Kit (perfect real time). Briefly, in the first step, cDNA was prepared from 1 *μ*g RNA by reverse transcription in a final volume of 20 *μ*L in an Opticon MJ Research instrument. The samples were incubated at 37°C for 60 min and 94°C for 5 min. The cDNA was stored at −20°C. The specific sense and antisense primers used were as follows: *α*-ENaC, sense: 5′-ACT GAA CTG TGC TCA GGG ATG A-3′, anti-sense: 5′-ATC TGC CTA CCT GGT CCA AGT G-3′; GAPDH, sense: 5′-CAG TGC CAG CCT CGT CTC AT-3′, anti-sense: 5′-AGG GCC TCC CAG CTT-3′. In the second step, quantitative real-time PCR was performed using the Opticon MJ Research instrument with SYBR Green from a SYBR GreenER qRT-PCR kit (perfect real time). The reaction was conducted with an initial denaturing at 94°C for 20 s, followed by 40 cycles of 60°C for 30 s and 72°C for 60 s, and then terminated by a cooling step at 4°C. A melting-curve analysis was performed to confirm the absence of primer dimers in the specific PCR products. The efficiency of the PCR was assessed with serial dilutions of a sample of cDNA from the normal control group. Each expression was performed in duplicate and the data were analyzed using the Opticon MJ Research Instrument Software.

### 2.8. Periodic Acid-Schiff (PAS) Staining

For morphological staining, isolated kidney tissues were fixed by immersion in 4% paraformaldehyde for 48 h at 4°C and then incubated with 30% sucrose for 2 days. Each kidney tissue sample was embedded in embedding medium, O.C.T. compound (Sakura Finetek USA Inc., Torrance, CA), frozen in liquid nitrogen, and then stored at −70°C until used in the experiments. Frozen sections were stained using the PAS reaction (Sigma, MO), according to the manufacturer's instructions as described by Sheehan and Hrapchak [25]. Slides were air-dried at room temperature for 30 min, rinsed in distilled water, and then rehydrated with 100%, 95%, 80%, and 70% alcohol for 5 min at room temperature and rinsed with distilled water for 5 min. Slides were incubated with periodic acid for 10 min at room temperature and rinsed with distilled water for 5 min. They were then incubated with Schiff's reagent for 5 min at room temperature and rinsed with distilled water for 5 min, followed by counterstaining with hematoxylin for 10 s, before being washed thoroughly in running tap water for 5 min. Slides were mounted with Balsam mounting medium and observed by microscopy (AxioVision 4, Zeiss, Germany).

### 2.9. Immunofluorescence Staining

Slides of the frozen kidney sections were fixed with 4% paraformaldehyde in phosphate-buffered saline (PBS) at room temperature for 15 min, after washing 3 times for 5 min in PBS. The slides were overlaid with 1% BSA for 1 h at room temperature in PBS and then incubated with primary antibodies for AQP2 (1 : 200; Santa Cruz Biotechnology, CA) overnight at 4°C. After washing, those were with rhodamine red-conjugated goat anti-rabbit IgG secondary antibody (Invitrogen, CA). The slides were incubated at room temperature for 1 h, and the apical plasma membrane was labeled by incubating cells with wheat germ agglutinin (WGA, Alexa Fluor 488 conjugate) (Invitrogen, Eugene, OR) diluted 1 : 500 in PBS. After washing 3 times, the sections were mounted with mounting medium and ProLong Gold Antifade Reagents (Molecular Probes, Eugene, OR) onto glass slides and observed by fluorescence microscopy (AxioVision 4, Zeiss, Germany).

### 2.10. Immunohistochemical Staining

Frozen sections to be used for immunohistochemical staining were immunostained using Histostain-SP kits according to the labeled-[strept]avidin-biotin (LABSA) method (Invitrogen Corporation, Carlsbad, CA). Frozen sections to be used for morphological staining were cut to a thickness of 10 *μ*m with a Shandon Cryotome SME and placed on noncoated slides. The slides were air-dried overnight at room temperature and stored at −70°C until morphological staining. Slides were observed by microscopy (AxioVision 4, Zeiss, Germany).

### 2.11. Statistical Analysis

Results were expressed as mean ± standard error of the mean. The statistical significant difference between the group means was determined using one-way analysis of variance (ANOVA) and Student's *t*-test. *P* < 0.05 was considered statistically significant.

## 3. Results

### 3.1. Effect of WPC on Body Weight, Urine Volume, Urine Osmolality, and Electrolytes

The mean body weight in the groups of PAN-induced nephrotic rats started at similar baseline levels. There were similar increases in the body weight in the Sham and the PAN groups (Table 1). Water intake was markedly increased in the PAN, Los, and WPC groups compared with that in the Sham group. Conversely, food intake increased in the control group but significantly decreased in the PAN, Los, and WPC groups (data not shown). Urine volume significantly increased in the PAN, Los, and WPC groups compared with the Sham group. Conversely, the urine osmolality significantly decreased in the PAN, Los, and WPC groups compared with the Sham group.

The electrolytes in the PAN group's urine significantly decreased compared with those in the Sham group. However, there was no difference in the levels of electrolytes between the WPC and the PAN groups (Table 1). Nevertheless, urine creatinine (Ucr), plasma creatinine (Pcr), and creatinine clearance (Ccr) were not markedly different in the PAN group compared with the Sham group (data not shown).

### 3.2. Effect of WPC on Plasma Biomarkers in Rats with PAN-Induced Nephrotic Syndrome

The total bilirubin level was not different in the PAN group when compared with the Sham or WPC groups. However, the Los group showed markedly decreased bilirubin levels in PAN-induced nephrotic rats. Plasma BUN levels were significantly lower (22%, *P* < 0.05) in the WPC group than in the PAN group. The total plasma protein levels decreased in the PAN group compared with the Sham group, whereas the WPC group showed a 12% increase compared with the PAN group (*P* < 0.05) (Table 2).

The blood glucose level of the PAN group showed no difference from that of the Sham and WPC groups. Significant increases in the plasma T-Cho, TG, and LDL-cholesterol levels were observed in the PAN group compared with the Sham group, whereas those in the WPC and Los groups were significantly lower than those in the PAN group. In contrast, HDL-cholesterol levels in the group treated with WPC were not different from those in the PAN group (Table 2). These results suggest that WPC could affect kidney function and cause lipidemia.

### 3.3. Effect of WPC on Kidney Dysfunction in Rats with PAN-Induced Nephrotic Syndrome

**Figure 1:**
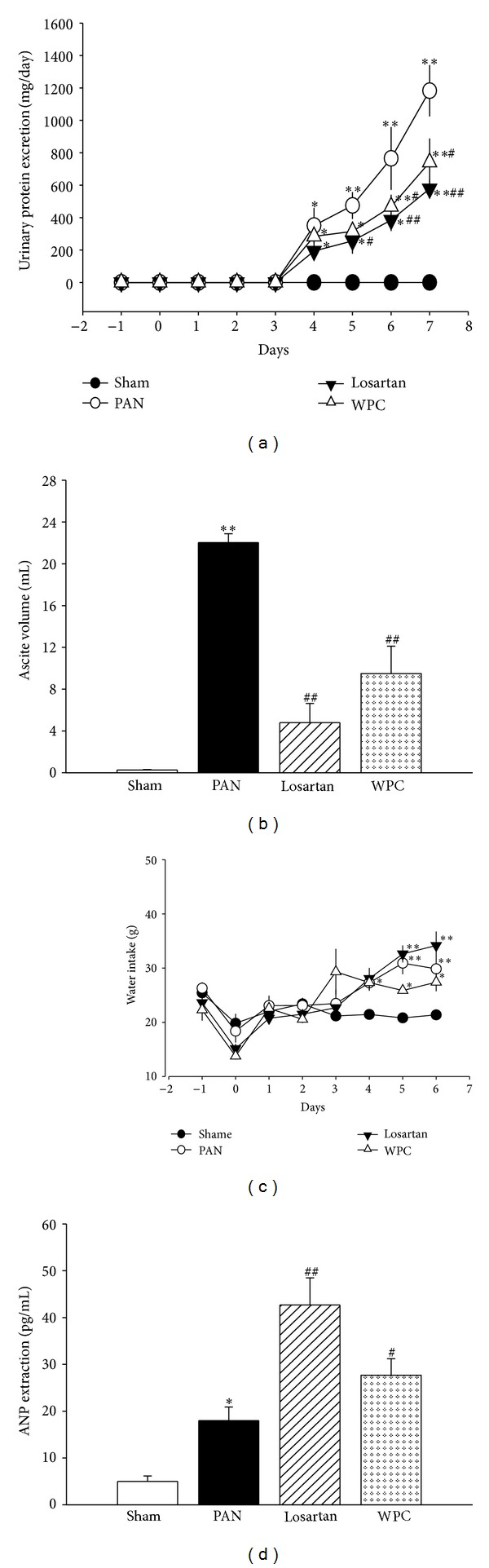
Effect of WPC on (a) urinary protein excretion, (b) ascites weight, (c) water intake, and (d) ANP extraction in PAN-induced nephrotic syndrome model rats. Rats received PAN (75 mg/kg), intraperitoneally, and then Losartan (30 mg/kg/day) or WPC (200 mg/kg/day) was administered. Values were expressed as mean ± S.E. (*n* = 5); *P < 0.05, **P < 0.05 versus Sham group; ^#^ *P* < 0.05, ^##^ *P* < 0.001 versus PAN group.

Higher urinary protein level was measured in the PAN group than in the Sham group. However, the WPC group showed markedly decreased urinary protein level. Similarly, the Los group exhibited remarkable decrease in urinary protein level compared to that in the PAN group (Figure 1(a)). Furthermore, PAN-injected rats developed severe nephrotic syndrome with marked ascites [26]. Therefore, we examined the effect of WPC on the inhibition of PAN-induced ascites.  Figure 1(b) shows that the PAN group had significantly increased ascites formation; however, in rats treated with WPC, there was 57% reduction in ascites formation (*P* < 0.001).

To explore the effect of WPC on the occurrence of plasma atrial natriuretic peptide (ANP), a comparison was made between the WPC and PAN groups, as determined by RIA. The plasma ANP levels in the PAN group increased compared with those in the Sham group. In addition, the plasma ANP levels in the WPC group significantly increased compared with those in the PAN group (Figure 1(c)).

### 3.4. Effect of WPC on Immunoreactivity for AQP2 Trafficking in the Renal Medulla

**Figure 2:**
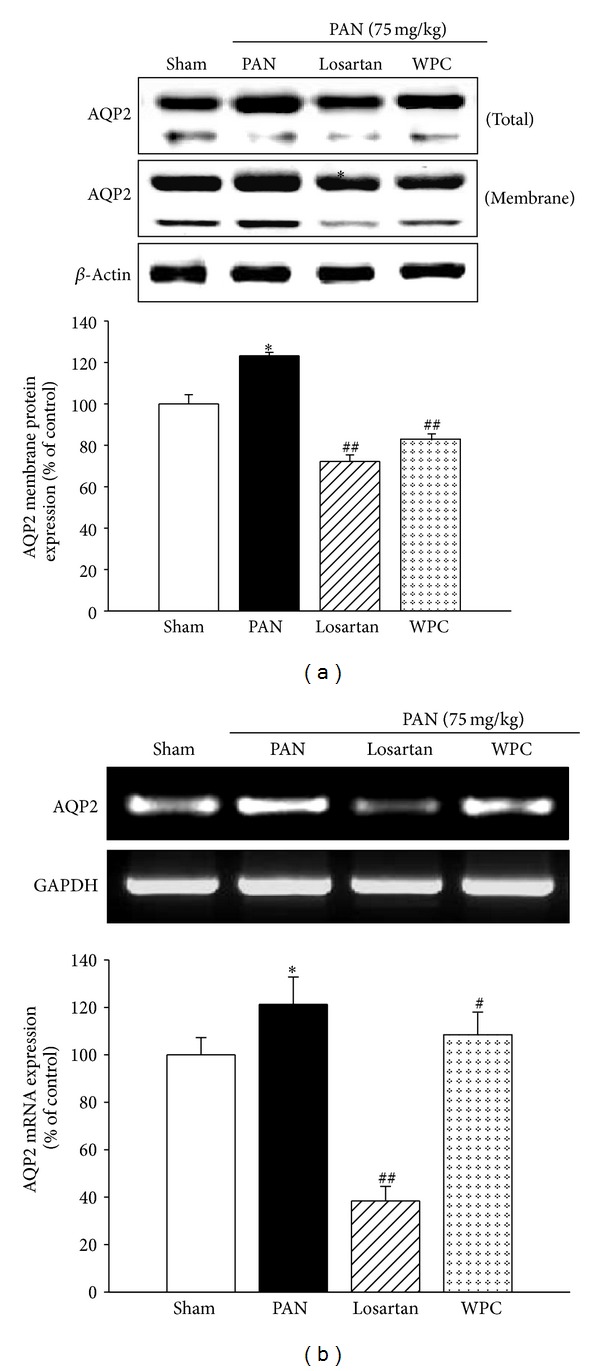
Effect of WPC on AQP2 protein and mRNA expression in PAN-induced nephrotic syndrome model. (a) The total protein and membrane fractions were extracted in medulla of kidneys and protein levels determined by Western blot analysis. (b) Total RNA was isolated from kidney of medulla, and then mRNA levels were determined by RT-PCR, respectively. The data are expressed as a percentage of basal value and are the means ± S.E. (*n* = 5); ^∗^ *P* < 0.05 versus Sham group; ^#^ *P* < 0.05, ^##^ *P* < 0.001 versus PAN group.

Western blot analysis for AQP2 expression was performed in the renal medulla. To determine whether WPC-induced decreases in AQP2 targeting affect the membrane insertion of AQP2, cytoplasmic and plasma membrane fractions were isolated. Compared with the Sham group, AQP2 total protein and membrane protein expression were markedly increased in the PAN group (Figure 2(a)). As shown, in Figure 2(b), the WPC group showed significantly reduced AQP2 protein and mRNA expression in the PAN-induced nephrotic syndrome model. AQP1 mRNA levels were also increased in the PAN group compared with those in the WPC group, but AQP3 did not show any difference (data not shown).

**Figure 3:**
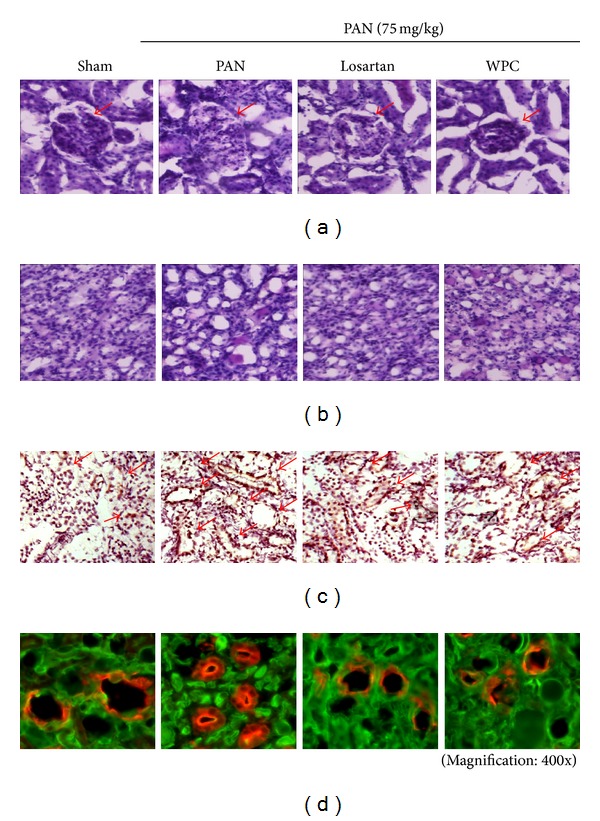
Effect of WPC ameliorates renal morphology changes and inhibition of AQP2 trafficking. (a) Periodic acid-Schiff (PAS) staining of the cortex and (b) inner renal medulla in rat kidney sections. (c) Immunohistochemistry staining of AQP2 (shown in brown) in the kidney medullar. Representative histological sections of kidney medulla of Sham group, untreated PAN group, nephrotic rat treated with Losartan group (30 mg/kg/day), and nephrotic rat treated with WPC group (200 mg/kg/day). (d) Immunofluorescence staining of AQP2 in renal medulla. AQP2 trafficking was stained in rhodamine (red). Membrane was visualized by WGA (green) (magnification: 400x).

The morphology of the renal medulla was observed before analyzing the immunoreactivity for AQP2 trafficking. Initially, the kidneys of rats with PAN-induced nephrotic syndrome were examined histopathologically. The PAN group showed increased glomerular and tubule epithelial enlargement, whereas the WPC group was markedly recovered (Figures 3(a) and 3(b)).

Acute regulation involves vasopressin-induced trafficking of AQP2 between an intracellular reservoir in the subapical vesicles and the apical plasma membrane [27]; thus, to determine the effects of WPC on AQP2 trafficking, we performed immunohistochemistry and immunofluorescence. Immunohistochemical staining analysis revealed that AQP2 was weakly expressed in the renal medulla of the Sham group but was markedly increased in the membranes of the collecting duct of the PAN group. In contrast, AQP2 expression was significantly decreased by treatment with WPC (Figure 3(c)). In addition, the tissue was then fixed, incubated with Alexa Fluor 488-tagged wheat germ agglutinin to mark the apical surface, permeabilized, and labeled with AQP2 (Red). Immunofluorescence staining of AQP2 indicated a markedly increased level in the PAN group compared with the Sham group; however, in the WPC group, AQP2 was significantly blocked (Figure 3(d)).

### 3.5. Effect of WPC on PAN-Induced ENaC Subunit Expression

**Figure 4:**
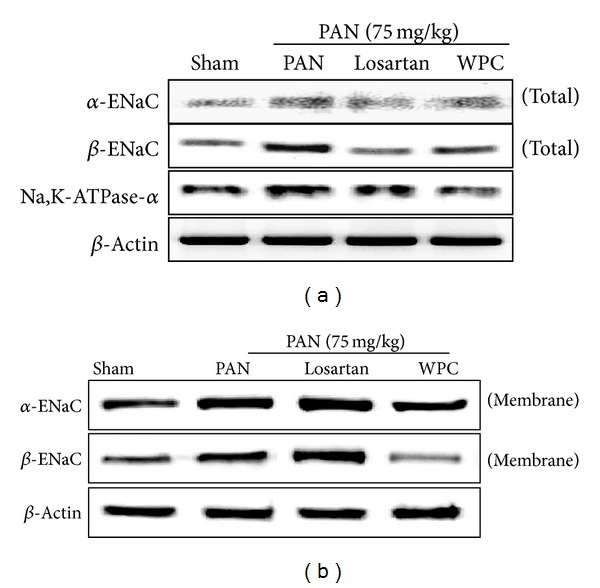
Effect of WPC on ENaC subunit and Na,K-ATPase-*α* protein expression in PAN-induced nephrotic syndrome model. (a) The kidneys of medulla were total protein extracted determined by Western blot analysis. (b) The membrane fractions were extracted in medulla of kidneys and protein levels were determined by Western blot analysis.

**Figure 5:**
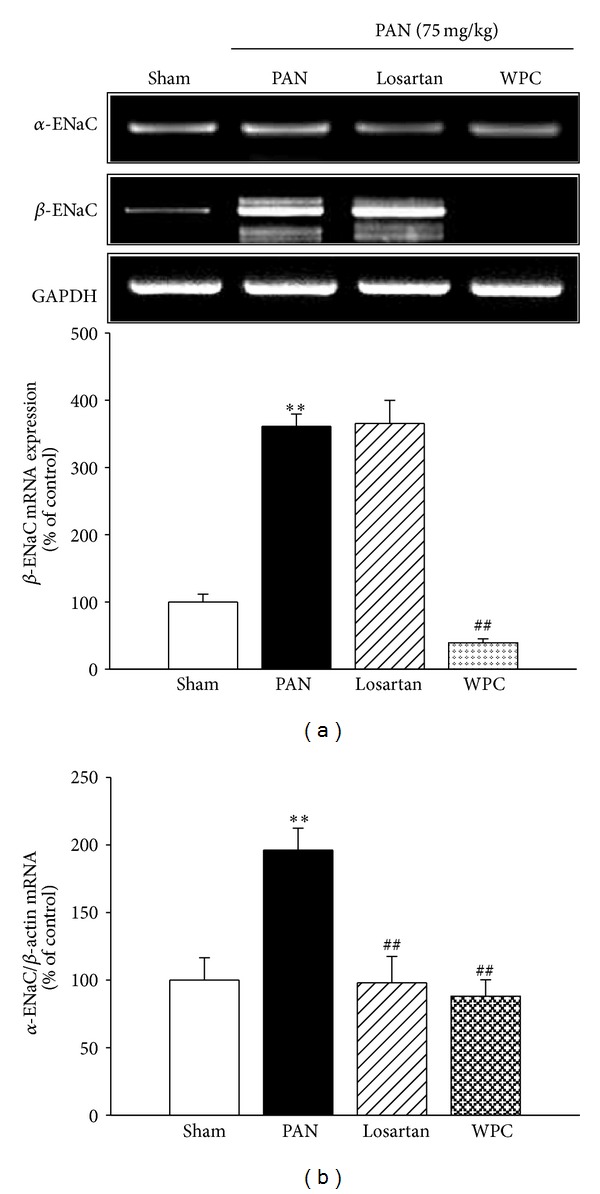
Effect of WPC on ENaC and MR mRNA levels expression in PAN-induced nephrotic syndrome model. (a) Total RNA was isolated from kidney of medulla, and then ENaC subunits mRNA levels were determined by RT-PCR, respectively. (b) Isolated total RNA was reverse-transcribed and *α*-ENaC mRNA was quantified using real-time PCR, as described in the methods section. Values were expressed as mean ± S.E. (*n* = 5); ^∗∗^ *P* < 0.001 versus Sham group; ^##^ *P* < 0.001 versus PAN group.

Experiments were conducted to determine whether ENaC subunit expression is altered in rats with PAN-induced nephrotic syndrome. The protein levels of the *α*-ENaC and *β*-ENaC subunits significantly increased in the PAN group. However, WPC evidently attenuated *α*-ENaC and *β*-ENaC protein expression in rats with PAN-induced nephrotic syndrome (Figure 4(a)). ENaC is also regulated by intracellular trafficking; therefore, to investigate whether the ENaC subunits are altered in rats with PNA-induced nephrotic syndrome, the plasma membrane fraction was examined. As shown, in Figure 4(b), a strongly enhanced apical membrane insertion of the *α*-ENaC and *β*-ENaC subunits was observed in the PAN group; however, the WPC group showed markedly decreased apical membrane *α*-ENaC and *β*-ENaC subunit insertion. In addition, the PAN group exhibited increased Na^+^/K^+^-ATPase *α*1 subunit; however, this was significantly decreased in the WPC group (Figure 4(a)). Furthermore, *α*-ENaC and *β*-ENaC mRNA levels significantly increased in the PAN group, as determined by RT-PCR. However, *α*-ENaC and *β*-ENaC subunit mRNA expression decreased in the WPC group (Figure 5(a)). In particular, the WPC group showed markedly decreased *α*-ENaC mRNA levels, as revealed by real-time RT-qPCR (Figure 5(b)). These data suggest that WPC may be involved in the regulation of sodium retention.

### 3.6. Effect of WPC on mRNA Expression of Serum- and Glucocorticoid-Inducible Kinase (Sgk1), Sodium-Myo-Inositol Transporter (SMIT), and Mineralocorticoid Receptor (MR)

**Figure 6:**
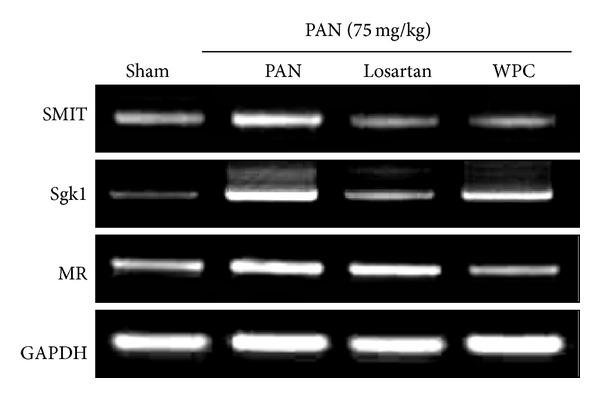
Effect of WPC on SMIT, Sgk1, and MR mRNA levels expression. Total RNA was isolated from kidney of medulla, and then SMIT, Sgk1, and MR mRNA levels were determined by RT-PCR, respectively.

Water reabsorption is associated with organic osmolyte regulators, such as Sgk1 and/or SMIT [28]. Furthermore, urinary protein excretion is the cause of the renal abundance of ENaC subunits via regulatory Sgk1 [29, 30].  Figure 6 shows that the mRNA level of Sgk1 was significantly upregulated in the PAN group compared with the Sham group, whereas that in the WPC group was markedly downregulated. SMIT mRNA level was also significantly affected in rats with PAN-induced nephrotic syndrome. Moreover, MR mRNA expression markedly increased in the renal medulla in the PAN group, whereas in the WPC group this was decreased.

## 4. Discussion

The results demonstrate that PAN-induced nephrotic syndrome is associated with decreased urine concentration, manifested by an increased urine output, decreased urine osmolality, and a marked upregulation of collecting duct water channels (AQP2) [31]. Furthermore, PAN-induced nephrotic syndrome correlates with sodium retention, decreased urinary excretion of sodium, marked ascites, and upregulation of protein levels of specific ENaC subunits [32]. The main symptoms of nephrotic syndrome are proteinuria, hypoproteinemia, sodium retention with edema formation, and hyperlipidemia [33–35]. This study induced nephrotic syndrome in rats by intraperitoneal injection of PAN (75 mg/kg).

Initially, in rats with PAN-induced nephrotic syndrome, kidney size might increase because of the damage from excessive urinary protein loss and glomerulosclerosis [36, 37]. In this study, the kidney weights of rats in the PAN group were significantly higher than those in the WPC group. It is postulated that WPC inhibits the renal hypertrophy developed because of the considerable protein level.

PAN-induced nephrotic syndrome causes hypoalbuminemia with increased plasma T-Chol and TG, and the albumin overload caused hyperalbuminemia with increased plasma-free fatty acids [38]. In a clinical trial in patients with nephrotic syndrome, urinary examination showed heavy proteinuria. The patients also had increased serum sodium concentrations and high plasma osmotic pressure, accompanied by high urinary osmotic pressure. Hypoproteinemia and serum cholesterol showed a marked decrease [39].

In this study, urinary protein excretion was higher in the PAN group than in the control group. However, administration of WPC significantly decreased protein excretion in rats with PAN-induced nephrotic syndrome. WPC is expected to improve renal fibrosis through inhibition of extracellular matrix accumulation in the glomerulus and reduced albuminuria. Furthermore, some studies have reported that hyperlipidemia is significant finding in nephrotic syndrome due to high serum TG and T-Chol levels [40, 41]. Nephrotic patients have a significantly lower blood volume and serum albumin than those in normal patients. Lipoprotein synthesis in the liver is increased to replace the lost plasma protein and lipoprotein carriers of TG and cholesterol. The serum level of TG in the PAN group was significantly higher than that in the Sham group. However, this was markedly decreased in the WPC group. In addition, the WPC group showed reduced levels of plasma T-Chol and LDL-cholesterol compared with those in the PAN group. Thus, it is considered that the hyperlipidemia in the PAN group is obviously due to the large portion of fat, whereas in the WPC group this was inhibited.

Ucr, Pcr, Ccr, and levels of plasma BUN are known indicators of renal dysfunction, and nephrotic syndrome progresses to renal functional impairment [42]. In this study, the levels of plasma BUN were increased in the PAN group, whereas they were significantly decreased in the WPC group; however, Ucr, Pcr, and Ccr levels showed no difference. Pilot studies preliminary to a full-scale study had shown increased Ucr and Pcr, contrary to this study, likely, because preliminary experimentation was conducted using a higher concentration of PAN (100 mg/kg). Thus, a low concentration of PAN may induce nephrotic syndrome, but have no influence on the levels of Ucr, Pcr, and Ccr.

In the present study, PAN-induced nephrotic syndrome is associated with decreased urine concentration, indicated by an increased urine output and natriuresis and decreased urine osmolality. Consistent with this, PAN-induced nephrotic syndrome is associated with a marked downregulation of AQP2 expression in the collecting duct [2]. Additionally, previous studies reported reduced collecting duct cell abundance of AQP2 in hypertonic conditions. This decrease plays an important role in regulating the water permeability of the collecting duct, with its level of concentration being associated with body-fluid regulation [15, 16]. In this study, it was found that, in rats with PAN-induced nephrotic syndrome, there were increases in the expression of AQP2 and urine output, whereas WPC significantly inhibited AQP2 trafficking, but urine output remained increased. Perhaps PAN-induced nephrotic syndrome involves increasing water intake and urine output, which causes an imbalance of water metabolism. However, WPC might inhibit the reabsorption of water and increase diuresis, which prevents the increase in body fluid and adjusts water metabolism. Further study is required to measure urinary antidiuretic hormone excretion relating to the inhibition of AQP2 expression and alleviation of kidney damage by WPC treatment in nephrotic syndrome.

It has recently been reported that there is an increase of ENaC activation in the kidney in PAN-induced nephrosis [32]. Furthermore, the expression and apical targeting of ENaCs have been shown to be increased in the collecting ducts in PAN-induced nephrotic syndrome and to be aldosterone dependent [43, 44]. Aldosterone plasma levels were not measured in the current study; however, it is presumed that aldosterone secretion occurred based on the increased MR observed in rats with PAN-induced nephrotic syndrome.

The interstitial edema observed in nephrotic syndrome results from primary renal sodium retention and secondary capillary fluid leakage. In PAN-induced nephrotic syndrome, renal sodium retention mainly originates from connecting tubules and collecting ducts [45]. In these nephron segments, sodium is reabsorbed by principal cells via passive apical entry through ENaCs and active basolateral extrusion by sodium pumps. The urine of patients with nephrotic syndrome contains active plasmin, which is the dominant serine protease in nephrotic urine, and can activate ENaC. Additionally, induction of nephrotic rats and human kidney leads to filtration of plasminogen into the urine, which is a urokinase-type plasminogen activator present in the rat and human kidney, and can convert inactive plasminogen to the active-form plasmin. Activation of ENaCs causes sodium retention, which is a major factor in edema formation in nephrotic syndrome [46–48]. Our findings suggest that induction of nephrotic syndrome in rats leads to activation of ENaC subunits, whereas WPC markedly inhibited the activity of ENaC subunits. On the basis of the damage to the glomerular filtration barrier, proteinuria, and stimulation of sodium, reabsorption was observed in distal tubules and collecting ducts in nephrotic syndrome [49]. PAN-induced nephrotic syndrome is also correlated with increased Na^+^/K^+^-ATPase activity and expression in the CCD [50]. The Na^+^/K^+^-ATPase *α*1 subunit also has specific effects in nephrotic syndrome; however, treatment with WPC attenuated Na^+^/K^+^-ATPase *α*1 subunit protein expression.

The present study also addressed the role of Sgk1 in the development of volume retention. A potential candidate is the Sgk1, which has originally been cloned as a glucocorticoid-inducible gene [51] and later as a cell volume-regulated gene [52]. Sgk1 is known to be upregulated by mineralocorticoids and is assumed to participate in the mineralocorticoid regulation of renal sodium excretion [53] and stimulate ENaCs [54], AQP2 [28], and Na^+^/K^+^-ATPase [55]. In the present study, Sgk1 mRNA expression was strongly enhanced in rats with PAN-induced nephrotic syndrome compared with normal rats, but the WPC group had an unaltered Sgk1 mRNA expression compared with that in rats with PAN-induced nephrotic syndrome.

In conclusion, the first implication of this study in rats with PAN-induced nephrotic syndrome is that the beneficial effect of WPC on water balance occurs via an inhibitory effect on PAN-induced AQP2 expression. WPC presumably blocks water reabsorption, resulting in body-fluid regulation. The second implication is that the reduced edema observed with WPC occurs via inhibition of ENaC activation. Thus, these results suggest that WPC suppresses AQP2 and ENaC expression and, therefore, improves the symptoms of nephrotic syndrome.

## Figures and Tables

**Table 1:** Effects of WPC on body weight and urine parameters.

	Body weight (g)		Urine volume (mL/kg/day)		Uosmol (osmol/kg)	
	Start	Final	Start	Final	Start	Final
Sham	195.58 ± 0.55	239.84 ± 4.87	27.83 ± 3.51	32.45 ± 3.64	5.38 ± 426.79	6.94 ± 623.57
PAN	195.92 ± 2.64	228.04 ± 5.22	35.52 ± 5.46	68.72 ± 19.00∗∗	7.42 ± 754.30	2.84 ± 1630.36∗∗
Losartan	201.62 ± 2.82	220.24 ± 4.21	29.42 ± 5.72	53.60 ± 9.97∗∗	6.83 ± 1376.62	2.30± 783.01∗∗
WPC	201.60 ± 1.77	230.18 ± 5.21	27.57 ± 4.66	56.95 ± 12.37∗∗	7.05 ± 1249.11	2.28 ± 994.74∗∗

	UNa^+^ (meq/24 h/kg)		UK^+^ (meq/24 h/kg)		UCl^−^ (meq/24 h/kg)	
	Start	Final	Start	Final	Start	Final

Sham	2.39 ± 102.84	2.15 ± 185.92	11.06 ± 989.83	13.48 ± 881.46	1.08 ± 1062.77	13.60 ± 967.50
PAN	2.17± 199.89	0.88 ± 135.24∗∗	12.38 ± 1198.84	10.52 ± 852.33∗	13.32 ± 1201.95	9.03 ± 2298.03∗∗
Losartan	2.48 ± 184.68	0.75 ± 133.15∗∗	10.83 ± 1239.06	9.55 ± 1347.23∗	12.30 ± 1844.92	8.39 ± 1719.22∗∗
WPC	2.58 ± 42.72	0.836 ± 81.45∗∗	10.74 ± 799.05	11.03 ± 452.83∗	12.73 ± 1109.83	9.64 ± 1448.94∗∗

Values are expressed as mean ± S.E. (*n* = 5); *P < 0.05, **P < 0.001 versus sham group.

**Table 2:** Effects of WPC on metabolic parameters.

	Sham	PAN	Losartan	WPC
Total bilirubin (mg/dL)	0.4 ± 0.1	0.5 ± 0.1	0.3 ± 0.1^#^	0.5 ± 0.1
BUN (mg/dL)	11.5 ± 1.1	43.4 ± 8.7**	59.4 ± 13.3	35.3 ± 0.6^#^
Total protein (g/dL)	5.4 ± 0.2	3.7 ± 0.2**	3.7 ± 0.2	3.9 ± 0.3^#^

Glucose (mg/dL)	130 ± 5.2	121.2 ± 7.9	127.6 ± 11.6	118 ± 8.4
Total cholesterol (mg/dL)	50 ± 0.1	359.8 ± 17.3**	258.7 ± 59.2	275.3 ± 20.6^#^
Triglyceride (mg/dL)	81.6 ± 14.1	446 ± 41.8**	178 ± 15.0^##^	264.7 ± 20.5^#^
HDL-c (mg/dL)	17.2 ± 1.3	85.8 ± 6.6**	117.3 ± 14.1^#^	89.2 ± 11.7
LDL-c (mg/dL)	16.5 ± 3.2	179.9 ± 15.4**	116.9 ± 20.6^#^	132.1 ± 8.1^#^

Values are expressed as mean ± S.E. (*n* = 5); **P < 0.001 versus control group; ^#^ *P* < 0.05, ^##^ *P* < 0.001, versus PAN group.
